# Grand Rounds: Outbreak of Hematologic Abnormalities in a Community of People Exposed to Leakage of Fire Extinguisher Gas

**DOI:** 10.1289/ehp.9197

**Published:** 2006-08-17

**Authors:** Shih-Hsiang Lo, Chang-Chuan Chan, Wei-Chin Chen, Jung-Der Wang

**Affiliations:** 1 Department of Internal Medicine, Zhongxing Branch of Taipei City Hospital, Taipei, Taiwan; 2 Institute of Occupational Medicine and Industrial Hygiene, College of Public Health, National Taiwan University, Taipei, Taiwan; 3 Department of Internal Medicine, National Taiwan University Hospital, Taipei, Taiwan; 4 Department of Environmental and Occupational Medicine, National Taiwan University Hospital, Taipei, Taiwan

**Keywords:** bromochlorodifluoromethane, bromotrifluoromethane, dichlorodifluoromethane, phosgene

## Abstract

**Context:**

Although there are ample data on the respiratory effects of exposure to fire extinguisher gas, the potential hematologic effects have not been fully documented. We conducted this study to determine the possible etiologic agent(s) for a decrease in red blood cells among community residents in Taipei, Taiwan, after they were exposed to leakage of mixed fire extinguishants containing bromotrifluoromethane (CF_3_Br, Halon 1301), bromochlorodifluoromethane (CF_2_BrCl, Halon 1211), and dichlorodifluoromethane (CCl_2_F_2_, CFC-12).

**Case presentation:**

We studied 117 exposed residents who came into one hospital for physical examinations. We also selected age- and sex-matched referents for comparison from residents who came to the same hospital for health examinations. Nine months after the exposure to mixed fire extinguishants, 91 of the exposed residents came back for a second physical examination. In the first examination of the exposed residents, we found a significant reduction in red blood cell count and hemoglobin and a relationship between dose and response.

**Discussion:**

After excluding iron-deficiency anemia, thalassemia, and other possible agents, we suspected that the hematologic effects might have resulted from pyrolytic products of CFC-12 and Halon 1211, which may contain phosgene, among other products.

**Relevance to clinical practice:**

The acute transient hematologic effects observed in the exposed residents were associated with the incident of leakage of mixed fire-extinguisher gases and were most likely caused by a small amount of pyrolytic products, probably phosgene. Nine months after the exposure, we found a significant improvement in the abnormalities without any specific treatment.

## Case Presentation

Carbon tetrachloride was first used extensively as a chemical fire extinguishant in the early 1920s. Since the 1950s, however, it has been replaced by less toxic halogenated compounds, mainly containing bromotrifluoromethane (CF_3_Br, Halon 1301) and bromochlorodifluoromethane (CF_2_BrCl, Halon 1211) (Bryan 1982). Unexpectedly, dichlorodifluoromethane (CFC-12, Freon-12, CCl_2_F_2_), a refrigerant, solvent, propellant, and fire extinguishant, was mixed with Halon gases in a fire-extinguishing agent in Taiwan.

Although acute exposure to Halon gases or CFC-12 has been reported to produce central nervous system symptoms [De La Hoz 1999; Harrison et al. 1982; Holness and House 1992; World Health Organization (WHO) 1990], respiratory tract symptoms (Brooks et al. 1985; De La Hoz 1999; Holness and House 1992; Kaufman et al. 1992), and cardiac arrhythmias (Holness and House 1992; Kaufman et al. 1992; Mullin et al. 1985), there have been no reports of hematologic changes related to these mixed fire extinguishants.

In the early morning of 8 May 2004 in the basement of Taipei City’s Department of Rapid Transit Systems’ Hua-Jie Transformer Substation, fire extinguisher gas was discharged to put out a small fire. The fire chief who first reached the scene reported blackish burned spots and maintenance tools on the floor, but no other substances burned. The gas from the fire extinguishers leaked to the external environment through a previously existing hole in the ceiling, which was about 8 m above the ground. The Bureau of Rapid Transit Systems reported a total of 1372.9 kg discharged fire extinguishants (228.8 kg/cylinder from six cylinders). Under low visibility, 152 residents ran out of their homes to escape the acidic, foul-smelling gas. The gas caused a variety of symptoms, particularly respiratory symptoms (cough, sore throat, and chest tightness), eye irritation, and light-headedness. Three of these residents were hospitalized due to severe chest discomfort. One was intubated for respiratory failure; fortunately, this resident recovered and was discharged from the hospital 3 days later.

**Figure 1:**
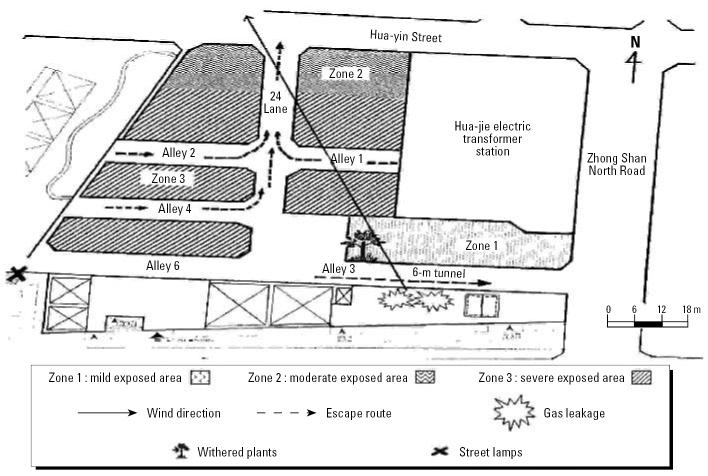
Geographical sketch of the exposed region showing wind direction and residents’ escape routes. Residents were classified into three exposure zones according to proximity to the leakage areas and exposure duration.

On 13 May 2004, all residents living in the exposed area (Figure 1) were invited by Taipei Bureau of Health to undergo a physical examination that included height, weight, blood pressure, heart rate, chest roentgenography, complete blood count, and liver function tests [including alanine aminotransferase (ALT) and aspartate aminotransferase (AST)] at the Zhongxing Branch of Taipei City Hospital. Of the community residents, 117 (77%) came voluntarily for the physical examination, and they all completed the physical examination during 14–27 May 2004, approximately 1 week after the exposure. Many were found to have a decrease in red blood cells (RBCs). Because abnormal hematologic effects have rarely been reported with mixed Halon exposure, we conducted this study to estimate the prevalence rates, determine the time courses of recovery, and identify the possible etiologic agent(s).

Nine months after the exposure, follow-up examinations were performed, including the same tests for hematology and biochemistry; 91 of the previously examined residents came for reexamination. Chest roentgenography and electrocardiography were also taken. Of these residents, 40 also underwent pulmonary function tests. The physical examination protocol was approved by the Hospital Review Board in Human Research, and all subjects who participated in the study provided written informed consent before physical examinations began.

### Questionnaires

The questionnaire portion of the study was conducted 9 months after the incident to examine the 117 residents who had been examined 1 week after the incident. Although the questionnaire was self-administered, researchers were available to answer any unclear questions.

The questionnaire asked participants to indicate which symptoms they had experienced before, during, and 9 months after the exposure (Table 1) (Holness and House 1992). To assess possible overreporting and recall bias, the questionnaire included three symptoms (abdominal pain, diarrhea, and muscle aches) that were anticipated to be uncommon in this situation.

### Nonexposed group

We compared the results from the exposed group with those of a nonexposed (control) group, which were recruited from 249 healthy subjects (≥ 15 years of age) who came to the same branch of Taipei City Hospital for regular screening examinations. These healthy subjects came from the same neighborhoods and were assessed during the same period of time, but they did not receive questionnaires and many of them also did not receive biochemical function tests. We matched the two groups by first stratifying the exposed residents into groups based on sex and 10-year age categories. We selected the same number of age- and sex-matched nonexposed residents using a random-digits table. Because we found no healthy controls to match the exposed residents < 15 years of age, we performed only a paired-comparison of hematology tests and biochemical function tests using data from the first week after exposure and 9 months after exposure.

### Exploring a potential dose–response effect

To determine the causal association between the exposure to mixed fire extinguishants and the hematologic effects, we explored the potential dose–response relationship. We conducted door-to-door interviews for the whole community and determined the exposure zones (shown in Figure 1) while blinded to the outcomes of physical examinations to avoid any possible bias. The residents in zone 1 reported that they were awakened in the middle of the night by a loud noise when the gas discharged; all of the residents in zone 1 escaped eastward through a narrow street (~ 6 m wide) within a few minutes. Residents in zones 2 and 3 all escaped northward, as shown in Figure 1. Zones 2 and 3 were located downwind from the leakage site, and a dense fog lasted almost 40 min in these two zones, according to the fire brigade’s archives. Because zone 2 was located 63 m from the leakage site, the residents were less exposed than those in zone 3. People in zone 3 generally took longer to escape than residents of zones 1 and 2, and thus received the highest exposure. For analyses, we combined zones 1 and 2 into the low-exposure group.

### Statistical analysis

We performed two sample *t*-tests to compare physical examination results of the exposed and nonexposed groups. To control for sex, we conducted multiple linear regressions using the nonexposed group as the reference; the nonexposed group was compared with the low-exposed (zones 1 and 2) and high-exposed (zone 3) subgroups. We then conducted a test for linear trend of dose–response relationship on each clinical parameter of hematology by assuming the levels of intensity of exposure for none (nonexposed), low (zones 1 and 2), and high (zone 3) as 0, 1, and 2, respectively for both sexes. Paired comparisons were analyzed by paired *t*-test for the exposed group; we compared results of physical examinations performed the first week and 9 months later, and used McNemar’s test to analyze hematologic data. These comparisons were performed to detect whether there were significant changes in the results of the health examinations during the period following the exposure to fire extinguisher gas. We further restricted the comparisons to subjects without thalassemia and/or iron-deficiency anemia to rule out other alternative causes. All statistical analyses were performed using SAS software (version 8.2; SAS Institute, Cary, NC, USA).

A total of 100 residents filled out the questionnaires (response rate of 66%). Of the questionnaires that were returned, eight were completed by the parents for younger children; five were filled out by family members on behalf of exposed residents because of illiteracy or poor vision; and the remaining questionnaires were filled out by the subjects themselves. Of the 17 who did not respond, 3 were mentally retarded, 7 had quit their jobs and/or moved from the community, and 7 refused to fill out the questionnaire for personal reasons. Many symptoms present within 1 week of the exposure were improved 9 months later (Table 1), but anxiety (41%) and sense of fear (54%) remained high.

Of the 117 residents who participated in the first week health examinations, 106 were > 15 years of age. The same number of age-and sex-matched nonexposed controls participated in the study. We found significant differences in the exposed and nonexposed groups for RBC count and hemoglobin (HB) in both sexes, as summarized in Table 2. Further stratifying the subjects by intensity of exposure, we found a significant linear trend between severity of exposure and reduction of RBCs and HB in both males and females. However, because of three male construction workers in zone 1 who were heavy alcohol drinkers, we observed mild elevations in both ALT and AST in the low-exposure subgroup, as shown in Table 2.

Of the residents, 91 (60%) returned for follow-up examination 9 months after the exposure. The paired comparisons of these 91 residents showed significant improvements for RBC counts, HB, and hematocrit (HCT), as summarized in Table 3. The differences in paired comparisons remained the same even after they were stratified by whether they were suspected of having thalassemia and/or iron-deficiency anemia. The 9 children < 15 years of age also showed the same improvements (data not shown). When we compared frequencies of abnormal hematologic results during the first week between those who returned for the follow-up and those who did not, we found no significant differences (data not shown). We used McNemar’s test to examine the difference in discordant pairs. RBC counts (*p* < 0.001), HB (*p* = 0.001), and HCT (*p* = 0.004) were statistically significant, indicating recovery from the exposure. The overall findings did show a consistent improvement for hematologic indicators 9 months later after controlling for potential confounding variables.

Moreover, at the health examination 9 months after exposure, 91 residents presented normal roentgenographic and electrocardiographic results. Their renal function tests, urine analyses, total bilirubin, direct bilirubin, and reticular cell counts were also within normal limits. All 40 of the subjects who had pulmonary function tests produced normal results.

## Discussion

In the first week after exposure, we found that residents exposed to fire-extinguisher gases had high prevalence rates of abnormal hematology findings or suspected anemia, which had returned to normal or improved 9 months later without any specific treatment. Although these findings alone do not necessarily indicate a causal relationship between the leaked gas exposure and abnormal hematologic tests, we hypothesize that such a relationship exists. First, there is a significant decrease in RBC count and HB in the exposed residents ⋛ 15 years of age compared with the age- and sex-matched nonexposed group during the same period (Table 2). The laboratory of the Zhongxin Branch of Taipei City Hospital has maintained excellent intralaboratory and inter-laboratory quality control/quality assurance programs. Thus, age, sex, or possible bias of laboratory tests cannot explain the difference. Second, when the exposed residents were divided into low- and high-exposure groups for comparison with the nonexposed group, the results showed a potential dose–response relationship for hematologic results, as summarized in Table 2. These hematologic indices improved, and more than half of the exposed residents completely recovered without any specific treatment (Table 3), fulfilling the necessary criteria of appropriate temporality (Rothman and Greenland 1998; Wang 2002). Third, after stratifying by young age (< 15 years) and by whether residents were suspected of having iron-deficiency anemia and/or thalassemia, thereby ruling out these confounders, we found consistent improvement in these abnormalities in our exposed group. Finally, we ruled out all other potential etiologic factors commonly listed in textbooks for anemia (Bloom and Brandt 2001; Brugnara and Lu 2003). Because > 80% of these residents suffered from respiratory symptoms the night they were exposed to the leaked fire extinguisher gases (8 May 2004), preexisting environmental airborne agents or factors may have been involved in their hematologic abnormalities. In addition, because they improved after 9 months, the outbreak of a specific disease does not fit the temporality criterion well. Therefore, we concluded that both the respiratory symptoms and hematologic effects were associated with this incident of leaked fire-extinguisher gases.

The results of the questionnaire indicated that the residents’ physical symptoms, including eye irritation and tears, cough, wheezing, nasal irritation, and sore throat, had improved by the ninth month after exposure. The prevalence rates of uncommon symptoms such as abdominal pain and diarrhea were 6%, suggesting the approximate magnitude of potential bias resulting from subjective report. Participants also reported persistent anxiety (41%) and sense of fear (54%), which may explain the high prevalence of somatic symptoms such as muscle ache (25%), fatigue (35%), and chest tightness (37%); 11 residents with somatic complaints came to the National Taiwan University Hospital for a more detailed examination, and none of them showed any additional sign or laboratory abnormalities. However, a potential selection bias is possible; because only 66% of the exposed residents filled out the questionnaire, the real prevalence of symptoms may be overestimated. To prevent subjective response bias of the questionnaires, we only showed the summary results of laboratory tests in the Table 2 and test for the dose–response trend. Although the respiratory symptoms of exposed residents were consistent with those in previous reports of Halon exposure (Brooks et al. 1985; De La Hoz 1999; Holness and House 1992; Kaufman et al. 1992), we wondered why there were decreases in RBC and HB.

The fire extinguisher system in the facility of the Rapid Transit System automatically releases the gases from a steel cylinder if both the smoke and local ambient temperature are detected > 60°C simultaneously. The Industrial Technology Research Institute (Hsinchu, Taiwan) examined all the steel cylinders and found that gas within them contained three kinds of halogenated carbons: Halon 1301, Halon 1211, and CFC-12. Samples taken from two cylinders previously filled showed that one of them contained 100% Halon 1301, but the other one contained a mixture of Halon 1211 (8.03%), Halon 1301 (1.75%), and CFC-12 (90.22%). Two of the samples taken from the six cylinders refilled after the leak contained mixed halogenated carbons: Halon 1211 (2.08 and 3.54%, respectively), Halon 1301 (63.52 and 55.45%), and CFC-12 (34.40 and 41.01%). These findings indicate that mixtures of halogenated carbons were added to pure Halon 1301 to make the gas in the cylinders.

**Figure 2:**
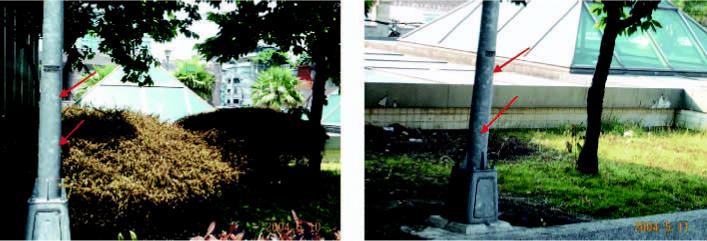
Whitish or corroded spots (arrows) were present on the metal surfaces of street lamp poles in the exposed region.

**Figure 3:**
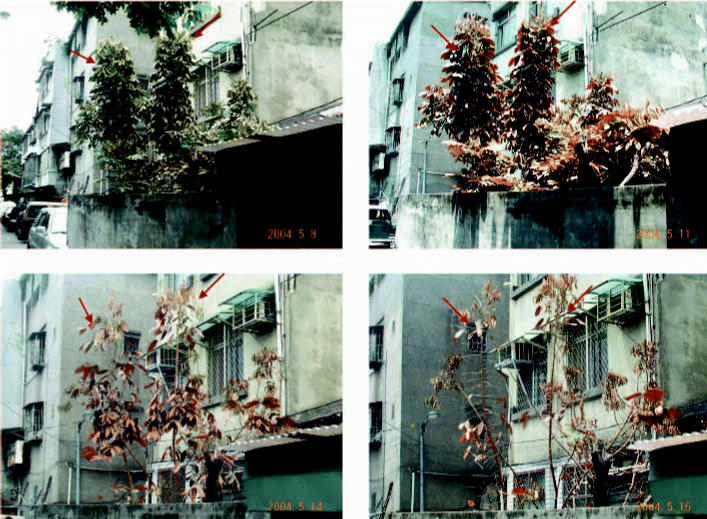
Serial photos showed yellowish discoloration of plants (arrows) exposed to gas leakage. Some plants (*Dimocarpus logan* shown here) withered and died.

Residents complained that the gas was acidic and foul-smelling the night of the exposure, which corroborates the hypothesis of mixed fire extinguishants with pyrolysis. In addition, pyrolytic products may have caused rusting and corrosion on the metal surfaces of street lamp poles and windows with metal bars in the exposed area, as shown in Figure 2. Several days after the incident, a yellowish discoloration appeared on plants in zone 1, and some of the plants withered and died, as shown in the Figure 3.

The pyrolytic products of Halon 1301 may contain hydrofluoric acid, hydrobromic acid, carbonyl-fluoride, and carbonyl-bromide, all of which have been reported as respiratory irritants but have not been reported to cause any hematologic effects (Harrison et al. 1982; Haun et al. 1996). However, the two chlorine atoms in CFC-12 and the one chlorine atom in Halon 1211 could produce phosgene (COCl_2_) (David and Neumann 1987; University of Michigan 2002) or related products if heated, as indicated by the following chemical reactions (Lanzafame 2005):


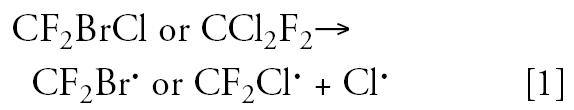



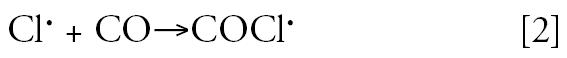



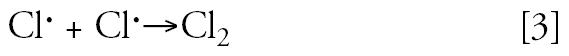



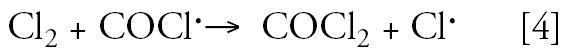


By assuming that the six containers of fire extinguishant contained about 37.5% CFC-12 by weight and that 1% of this CFC-12 underwent pyrolytic reaction during the fire, we estimated that approximately 1.76 g/sec phosgene was emitted for 40 min, with a bulk property of dense gas (i.e., gas denser than the air). By applying the dense gas dispersion models (Louvar and Louvar 1998) to phosgene emitted from the Hua-Jie Transformer Substation’s roof with a wind speed of 2 m/sec, we calculated that ground-level phosgene concentrations were about 151 ppm at 30 m downwind, and 39 ppm at 60 m downwind, the distances covered for zones 2 and 3.

In animal studies (guinea pigs, rats, and mice), Sciuto et al. (1996) reported that whole-body exposure to phosgene (> 22 ppm, 20 min) resulted in alterations of red blood cell membranes with increased fragility and increased hemolysis. Small amounts of phosgene dissolved in water produce hydrochloric acid, an eye and throat irritant (Borak and Diller 2001); this seems to corroborate the acidic, foul odor that exposed residents reported smelling that night. Because none of the hospitalized exposed residents showed unequivocal evidence of pulmonary edema, the level of phosgene, if produced, may be < 150 ppm, which is compatible with the hypothesis of low-level exposure (Borak and Diller 2001; Bradley and Unger 1982; Diller 1978, 1985; Wells 1985) and the above calculation. As we rule out agents ever reported to cause anemia, phosgene generated from pyrolysis of mixed fire extinguishants seems to be the most likely cause of the acute transient anemia in the exposed residents.

Because we did not pursue the hematologic results of the initial examination to determine if the anemia was hemolytic or caused by marrow injury at the time of first occurrence, we were unable to make any strong inference. Based on our findings and precautionary principles, we suggested that more studies should be conducted to corroborate the possible etiologic agent(s) if chlorine-containing CFCs are still used in fire extinguishants. Moreover, any area exposed to Halon gasses (e.g., areas where fires have been extinguished) should be closed off from the outside environment. Once the fire is under control, the gases should be slowly pumped out and diluted with air in the atmosphere while preventing direct exposure to the local residents.

## Conclusion

We conclude that acute effects of respiratory and other physical symptoms, as well as transient reduction of RBCs and HB, in the exposed residents were associated with the incident of leaked mixed fire extinguisher gases. Although the respiratory and other physical symptoms may be caused by mixtures of Halon 1301, CFC-12, and Halon 1211 and/or their pyrolytic products, the hematologic effects were most likely caused by the small amount of their pyrolytic products, probably phosgene. The symptoms related to anxiety and sense of fear still remained high in residents even at the 9 month follow-up. The residents in the exposed area should be followed up further to ensure that they have no residual long-term effects from the exposure to mixed fire-extinguisher gases.

## Figures and Tables

**Table 1:** Frequencies (%) of symptoms among the 100 exposed residents before, during, and 9 months after exposure to mixed fire extinguishants.

Symptom	Before exposure	During exposure	9 Months after exposure
Eye irritation	0.02	0.58	0.25
Cough	0.03	0.80	0.38
Wheezing	0.01	0.17	0.13
Sneeze and rhinorrhea	0.02	0.32	0.25
Sore throat	0.02	0.69	0.20
Voice change	0.01	0.30	0.11
Chest tightness	0.01	0.64	0.37
Chest pain	0.01	0.31	0.15
Palpitation	0.02	0.30	0.22
Shortness of breath	0.00	0.14	0.15
Tachypnea	0.02	0.36	0.14
Light-headedness	0.01	0.50	0.24
Headache	0.00	0.31	0.16
Unstable gait	0.01	0.22	0.10
Unconsciousness	0.00	0.17	0.07
Fatigue	0.03	0.39	0.35
Nausea	0.00	0.40	0.10
Vomiting	0.03	0.33	0.11
Numbness of fingers	0.01	0.06	0.12
Anxiety	0.04	0.48	0.41
Sense of fear	0.00	0.69	0.54
Skin itch	0.02	0.12	0.17
Skin blister	0.00	0.02	0.04
Abdominal pain^a^	0.01	0.06	0.09
Diarrhea^a^	0.01	0.06	0.09
Muscle pain^a^	0.02	0.23	0.25

a Uncommon symptoms after exposure to fire extinguishants.

**Table 2:** Comparison of clinical test results between exposed residents (initial physical examination) and the nonexposed community residents.

	Female					Male				
	Exposure					Exposure				
	None (*n* = 57)	Low (*n* = 17)	High (*n* = 40)	Total exposed^a^ (*n* = 57)	Test for linear trend	None (*n* = 49)	Low (*n* = 22)	High (*n* = 27)	Total exposed^a^ (*n* = 49)	Test for linear trend
Age [mean (1 SD)]	43.77 (16.77)	47.26 (17.78)	41.31 (18.61)	43.09 (18.42)		44.39 (19.02)	43.36 (18.02)	44.39 (17.97)	43.93 (17.81)	
Hematologic tests [mean (1 SD)]										
WBC (× 10^3^/μL)	6.1 (2.22)	6.08 (1.58)	6.22 (1.62)	6.18 (1.60)	0.76	6.81 (2.03)	6.39 (1.38)	6.71 (1.62)	6.57 (1.51)	0.829
RBC (× 10^6^/μL)	4.52 (0.47)	4.22 (0.40)	4.2 (0.50)	4.21 (0.47)**	0.001	5.07 (0.50)	4.73 (0.34)	4.66 (0.47)	4.69 (0.42)**	< 0.001
HB (g/dL)	12.98 (1.23)	12.71 (1.12)	12.39 (1.00)	12.48 (1.04)*	0.011	15.18 (1.14)	14.71 (1.03)	14.16 (1.13)	14.40 (1.11)**	< 0.001
HCT (%)	39.21 (3.30)	37.67 (2.96)	38.49 (9.95)	38.25 (8.46)	0.508	44.66 (2.73)	42.67 (2.50)	42.83 (4.88)	42.76 (3.95)*	0.149
MCV (fL)	87.31 (7.85)	89.61 (6.29)	88.72 (8.69)	88.98 (8.00)	0.381	88.74 (7.83)	90.37 (5.09)	89.99 (8.48)	90.16 (7.09)	0.503
MCH (pg)	28.92 (3.00)	30.29 (2.77)	29.73 (3.71)	29.9 (3.44)	0.212	30.18 (2.96)	31.14 (2.17)	30.65 (3.56)	30.87 (3.00)	0.528
MCHC (g/dL)	33.09 (0.86)	33.73 (1.13)	33.52 (1.17)	33.58 (1.15)*	0.047	33.98 (0.98)	34.44 (1.06)	33.83 (1.29)	34.1 (1.22)	0.564
PLT (× 10^3^/μL)	252.35 (51.40)	250.18 (70.44)	222.6 (54.89)	230.82 (60.64)*	0.006	226.1 (39.14)	211.5 (45.31)	219.41 (59.67)	215.86 (53.32)	0.591
Biochemical function tests [mean (1 SD)]^b^										
BUN (mg/dL)		12.76 (3.54)	14.33 (3.63)	13.86 (3.65)			15.23 (4.58)	17.56 (5.98)	16.51 (5.47)	
AST (U/L)		16.65 (5.59)	17.95 (7.80)	17.56 (7.19)			26.5 (20.56)	23.37 (10.30)	24.78 (15.65)	
ALT (U/L)		11.41 (6.31)	14.03 (15.64)	13.23 (13.49)			29.73 (38.61)	27.48 (18.88)	28.49 (29.09)	

Abbreviations: BUN, blood urea nitrogen; MCH, mean cell HB; MCHC, mean cell HB concentration; MCV, mean cell volume; PLT, platelets.

a Exposed compared with nonexposed in the same strata by two-sample *t*-test.

b No biochemical function tests were available from the nonexposed group.

* *p* < 0.05.

** *p* < 0.005.

**Table 3:** Comparison of hematology and biochemical function tests in 91 exposed residents within 1 week and 9 months after fire-extinguisher gas exposure.

	1 Week after exposure (mean ± SD)	9 Months after exposure (mean ± SD)	Mean difference (95% CI)^a^	No. of abnormal cases^b^	No. of cases recovered or improved^c^
RBC (× 10^6^/μL)	4.35 ± 0.45	4.61 ± 0.47	0.26 (0.21–0.31)	50	43
HB (g/dL)	13.34 ± 1.38	13.99 ± 1.38	0.65 (0.51–0.79)	29	27
HCT (%)	39.49 ± 4.53	41.45 ± 3.83	1.96 (1.26–2.66)	36	34
MCV (fL)	90.28 ± 5.94	90.24 ± 5.96	−0.04 (^−^0.44–0.52)	5	3
MCH (pg)	30.80 ± 2.42	30.49 ± 2.38	−0.31 (0.13–0.49)	5	1
MCHC (g/dL)	34.04 ± 1.01	33.76 ± 0.99	−0.28 (0.14–0.42)	7	2
WBC (× 10^3^/μL)	6.31 ± 1.51	6.11 ± 1.56	−0.49 (^−^0.11–0.50)	12	7
PLT (× 10^3^/μL)	226.69 ± 55.75	236.07 ± 52.18	9.37 (3.78–14.97)	1	0
BUN (mg/dL)	15.53 ± 4.74	14.12 ± 4.23	−1.41 (0.50–2.31)	2	0
AST (U/L)	20.22 ± 8.19	22.40 ± 9.55	2.18 (1.05–3.30)	7	0
ALT (U/L)	17.24 ± 14.13	22.68 ± 19.35	5.44 (3.51–7.37)	7	1

Abbreviations: BUN, blood urea nitrogen; CI, confidence interval; MCH, mean cell HB; MCHC, mean cell HB concentration; MCV, mean cell volume; PLT, platelets.

a Paired comparison within every subject.

b Number of abnormal cases in the first examinations.

c Number of cases recovered or improved 9 months later.
